# Single Chelator–Minibody Theranostic Agents for ^89^Zr PET Imaging and ^177^Lu Radiopharmaceutical Therapy of PSMA-Expressing Prostate Cancer

**DOI:** 10.2967/jnumed.124.267667

**Published:** 2024-09

**Authors:** Khanh-Van Ho, David S. Tatum, Lisa Watkinson, Terry Carmack, Fang Jia, Alessandro Mascioni, Charles A. Maitz, Darren Magda, Carolyn J. Anderson

**Affiliations:** 1 Department of Chemistry, University of Missouri, Columbia, Missouri;; 2 Molecular Imaging and Theranostics Center, University of Missouri, Columbia, Missouri;; 3 Lumiphore Inc., Berkeley, California;; 4 Research Service, Harry S. Truman Memorial Veterans’ Hospital, Columbia, Missouri;; 5 ImaginAb Inc., Inglewood, California;; 6 Department of Veterinary Medicine and Surgery, University of Missouri, Columbia, Missouri;; 7 MU Research Reactor, University of Missouri, Columbia, Missouri;; 8 Department of Radiology, University of Missouri, Columbia, Missouri; and; 9 Ellis Fischel Cancer Center, University of Missouri, Columbia, Missouri

**Keywords:** L804, minibody, theranostics, targeted radiotherapy, prostate cancer

## Abstract

Here we describe an anti–prostate-specific membrane antigen (PSMA) minibody (IAB2MA) conjugated to an octadentate, macrocyclic chelator based on four 1-hydroxypyridin-2-one coordinating units (Lumi804 [L804]) labeled with ^89^Zr (PET imaging) and ^177^Lu (radiopharmaceutical therapy), with the goal of developing safer and more efficacious treatment options for prostate cancer. **Methods:** L804 was compared with the current gold standard chelators, DOTA and deferoxamine (DFO), conjugated to IAB2MA for radiolabeling with ^177^Lu and ^89^Zr in cell binding, preclinical biodistribution, imaging, dosimetry, and efficacy studies in the PSMA-positive PC3-PIP tumor–bearing mouse model of prostate cancer. **Results:** Quantitative radiolabeling (>99% radiochemical yield) of L804-IAB2MA with ^177^Lu or ^89^Zr was achieved at ambient temperature in under 30 min, comparable to ^89^Zr labeling of DFO-IAB2MA. In contrast, DOTA-IAB2MA was radiolabeled with ^177^Lu for 30 min at 37°C in approximately 90% radiochemical yield, requiring further purification. Using europium(III) as a luminescent surrogate, high binding affinity of Eu-L804-IAB2MA to PSMA was demonstrated in PC3-PIP cells (dissociation constant, 4.6 ± 0.6 nM). All 4 radiolabeled constructs showed significantly higher levels of internalization after 30 min in the PC3-PIP cells than in PSMA-negative PC3-FLU cells. The accumulation of ^177^Lu- and ^89^Zr-L804-IAB2MA in PC3-PIP tumors and all organs examined (i.e., heart, liver, spleen, kidney, muscle, salivary glands, lacrimal glands, carcass, and bone) was significantly lower than that of ^177^Lu-DOTA-IAB2MA and ^89^Zr-DFO-IAB2MA at 96 and 72 h after injection, respectively. Generally, SPECT/CT and PET/CT imaging data showed no significant difference in the SUV_mean_ of the tumors or muscle between the radiotracers. Dosimetry analysis via both organ-level and voxel-level dose calculation methods indicated significantly higher absorbed doses of ^177^Lu-DOTA-IAB2MA in tumors, kidney, liver, muscle, and spleen than of ^177^Lu-L804-IAB2MA. PC3-PIP tumor–bearing mice treated with single doses of ^177^Lu-L804-IAB2MA (18.4 or 22.2 MBq) exhibited significantly prolonged survival and reduced tumor volume compared with unlabeled minibody control. No significant difference in survival was observed between groups of mice treated with ^177^Lu-L804-IAB2MA or ^177^Lu-DOTA-IAB2MA (18.4 or 22.2 MBq). Treatment with ^177^Lu-L804-IAB2MA resulted in lower absorbed doses in tumors and less toxicity than that of ^177^Lu-DOTA-IAB2MA. **Conclusion:** ^89^Zr- and ^177^Lu-L804-IAB2MA may be a promising theranostic pair for imaging and therapy of prostate cancer.

## Visual Abstract

**Figure:**
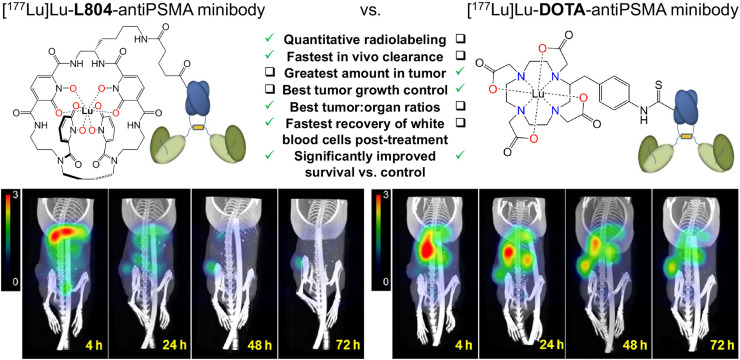

Although only recently approved, small-molecule constructs targeting prostate-specific membrane antigen (PSMA), an exceptional biomarker for prostate cancer, have achieved extraordinary clinical success as PET imaging agents when labeled with ^18^F (^18^F-DCFPyL [Pylarify; Lantheus]) (1) or ^68^Ga (^68^Ga-PSMA-11 [Illucix; Telix Pharmaceuticals]/[Locametz; Novartis]) (2,3). A therapeutic counterpart using ^177^Lu (^177^Lu-PSMA-617 [Pluvicto; Novartis]) was approved in March 2022 after demonstrating a clear survival benefit (median, 4 mo) over the standard of care for patients with heavily pretreated metastatic castration-resistant prostate cancer (4), and additional development of analogous small-molecule agents is ongoing (5,6). However, small-molecule PSMA-binding agents also localize to the salivary glands, which can be a significant source of off-target toxicity for the therapeutic agents (7)—notably dose-limiting for α-emitter constructs such as ^225^Ac-DOTA-PSMA-617 (8,9). In contrast, PSMA-targeting antibody-based constructs do not localize significantly to the salivary glands, and so these biologic agents may provide a path toward safer radiotherapeutic options for prostate cancer (10,11). Antibody-based agents such as ^177^Lu-DOTA-J591 have been shown to suffer from hematopoietic dose-limiting toxicity challenges (i.e., myelosuppression) (12–14). Nevertheless, ^177^Lu-DOTA-J591 has recently entered late-stage testing (NCT04876651) on a ^68^Ga-PSMA-11 prescreened patient population following a fractionated dosing scheme to mitigate this dose-limiting toxicity.

The bone marrow toxicity observed for ^177^Lu-DOTA-J591 is thought to arise from at least 2 potential sources. First, the slower clearance rate of antibody constructs from blood circulation is thought to innately contribute to increased toxicity (15). Second, antibody constructs cannot be heated to the temperatures necessary for complete incorporation of ^177^Lu into the DOTA chelator (16,17), and yet their long circulation times necessitate even greater chelate stability than for rapidly excreted small-molecule agents. Incorporation of trivalent rare earth metal ions into DOTA proceeds slowly through 2 out-of-cage intermediates, necessitating high temperatures to form the in-cage complex (18). The directly radiolabeled ^177^Lu-DOTA-antibody constructs may suffer from significant out-of-cage binding of ^177^Lu, a condition for which discrimination via a convenient analytic technique does not exist to the best of our knowledge (19,20).

Here we investigated the effects of 2 potential improvements to ^177^Lu-DOTA-J591. First, we made use of a minibody (80 kDa) derivative of J591 (IAB2MA; ImaginAb Inc.). The related ^89^Zr-DFO-IAB2M minibody construct exhibited faster clearance rates and earlier peak signal-to-background ratios than did the analogous ^89^Zr-DFO-J591 antibody construct (11,21). Compared with IgG antibodies, minibodies generally exhibit faster clearance and earlier tumor accumulation, and the Fc receptor–mediated interactions have been eliminated (22,23). Second, we evaluated a recently developed macrocyclic chelator, Lumi804 (L804; Lumiphore Inc.), which can be radiolabeled with ^177^Lu (and ^89^Zr) at room temperature and offers a stability advantage over the current state-of-the-art DOTA (and DFO) chelators.

The biodistribution, therapeutic efficacy, SPECT imaging, and dosimetry (using 2 methods: based on organ-level and voxel-level dose calculations) of ^177^Lu-L804-IAB2MA and ^177^Lu-DOTA-IAB2MA were compared in the PSMA-expressing (PC3-PIP) prostate tumor–bearing mouse model, along with the biodistribution and PET imaging of ^89^Zr-L804-IAB2MA and ^89^Zr-DFO-IAB2MA. In addition to the potential advantages for any one radionuclide, the L804 chelator binds both ^177^Lu and ^89^Zr exceptionally well, as demonstrated previously (24). In contrast, ^89^Zr labeling of DOTA is challenging (25,26), and ^177^Lu-labeled DFO lacks sufficient in vivo stability. The comparison of murine-model performance between L804 and DFO antibody constructs labeled with ^89^Zr has been previously reported, and the advantage of L804 was found to be most apparent in decreased bone radioactivity at later time points (24). L804 has also been recently compared with DOTA and other chelators for ^227^Th and ^89^Zr labeled ofatumumab constructs (27). This report represents the first direct comparison of L804 and DOTA constructs with ^177^Lu, using a well-validated cancer target and an improved biologic vector, with the aim of improving treatment and imaging of prostate cancer.

## MATERIALS AND METHODS

Details on reagents (28), animal husbandry, minibody construct preparation, radiolabeling, Eu-L804-IAB2MA binding assays (24), radiolabeled internalization assays (29), and statistical analysis are included in the supplemental methods (supplemental materials are available at http://jnm.snmjournals.org). All animal experiments were conducted in compliance with the Institutional Animal Care and Use Committee at the University of Missouri.

### Cell Lines and Mouse Xenografts

PC3 wild-type (also called FLU) human prostate cancer cells (CRL-1435) were purchased from American Type Culture Collection. PC3-PIP human prostate cancer cells were a gift from Johns Hopkins University. Cells were maintained in 45% RPMI 1640, 45% Ham F-12, and 10% heat-inactivated fetal bovine serum. For xenograft tumors, mice were subcutaneously implanted with 0.5 × 10^6^ PC3-PIP cells (0.5 × 10^7^ cells/mL) or 1 × 10^6^ PC3-FLU cells (1 × 10^7^ cells/mL) in the axilla (under the forelimb).

### Biodistribution of Radiolabeled Constructs

At 21 d after implantation of PC3-PIP prostate cancer cells, the mice were injected via tail vein with ^89^Zr-L804-IAB2MA, ^89^Zr-DFO-IAB2MA, ^177^Lu-L804-IAB2MA, or ^177^Lu-DOTA-IAB2MA and euthanized at time points from 4 to 96 h afterward, with blocking dose groups at 24 h (4 mice per group per time point). For the ^89^Zr agents, each mouse received 100 μL of either 0.185 MBq (4, 24, and 48 h) or 0.37 MBq (72 h) of ^89^Zr-L804-IAB2MA or ^89^Zr-DFO-IAB2MA at a molar activity of 1.85 MBq/nmol. For the ^177^Lu agents, each mouse received 100 μL of either 0.185 MBq (4 and 24 h) or 0.74 MBq (48, 72, and 96 h) of ^177^Lu-L804-IAB2MA or ^177^Lu-DOTA-IAB2MA at a molar activity of 7.4 MBq/nmol. Mice preloaded with unlabeled minibody (10 mg/kg) before the injection of the radiotracers were used as blocking control groups. Biodistribution of all radiotracers was also performed on PC3-FLU tumor–bearing mice at 24 h after injection (supplemental methods). At each time point, organs were collected and weighed. Tissue-associated radioactivity was determined in an automatic γ-counter (1480 Wizard 3″; PerkinElmer) and is expressed as percentage injected dose (%ID)/g.

### PET/CT and SPECT/CT Imaging

PET/CT and SPECT/CT imaging studies (Albira Si; Bruker) were performed on PC3-PIP tumor–bearing mice at 21 d after implantation. The mice were injected via the tail vein with 100 μL of ^89^Zr-L804-IAB2MA or ^89^Zr-DFO-IAB2MA (5.55 MBq) at a molar activity of 9.25 MBq/nmol or with ^177^Lu-L804-IAB2MA or ^177^Lu-DOTA-IAB2MA (22.2 MBq) at a molar activity of 18.5 MBq/nmol (3 male mice per group). For PET imaging, mice injected with the ^89^Zr agents were scanned at 4, 24, and 48 h after injection. For the SPECT/CT imaging, mice injected with the ^177^Lu agents were scanned at 4, 24, 48, and 72 h after injection. Regions of interest were drawn using the CT scan, and the associated PET activities were calculated using Imalytics Preclinical 3.1 (Gremse-IT) (30) and expressed as %ID/g.

### Dosimetry

Absorbed doses were determined using 2 different internal dose calculation software platforms. MIRDCalc 1.2 (31,32) was used to determine absorbed doses (Gy/MBq) in tumors and organs (i.e., kidney, liver, heart, spleen, and salivary glands) obtained from the biodistribution study of ^177^Lu-L804-IAB2MA and ^177^Lu-DOTA-IAB2MA at 4, 24, 48, and 72 h after injection. Imalytics was used to calculate absorbed doses in tumors and organs (i.e., kidney, liver, lung, and muscle) from the SPECT/CT imaging data of ^177^Lu-L804-IAB2MA and ^177^Lu-DOTA-IAB2MA at 4, 24, 48, and 72 h after injection (supplemental methods).

### Targeted Radiopharmaceutical Therapy

At 9 d after implantation of PC3-PIP tumor cells (1 × 10^7^ cells per mouse) in the right flank, the mice were randomized to provide an average tumor volume of approximately 6 mm^3^ and an average body weight of approximately 24 g in each group (8 male mice). The mice received single therapeutic doses of ^177^Lu-L804-IAB2MA or ^177^Lu-DOTA-IAB2MA (14.8 and 22.2 MBq; 100 μL; 18.5 MBq/nmol). Non–tumor-bearing mice were included as a control group (blood control, 8 male mice). An additional control group (tumor-bearing mice) received unlabeled minibody (2.2 mg/kg). Tumor volumes were measured (length × width × height), and the mice were monitored twice weekly for weight loss. Blood was drawn by tail vein and was collected into ethylenediaminetetraacetic acid–coated tubes at 2 d before the treatment and every 2 wk thereafter for complete blood count analysis (VetScan HM5 hematology analyzer; Abaxis). Mice that were found in a moribund condition, had at least 20% body weight loss, or had a tumor burden of at least 1,500 mm^3^ reached the endpoint and were removed from the study. At the time of euthanasia, all major organs were harvested and analyzed for signs of radiotoxicity. Data were plotted as a Kaplan–Meier graph of time when the mice were removed from the study.

## RESULTS

### Synthesis and Radiolabeling Chemistry

Chelator-to-minibody ratios of 2–2.5 were achieved for all conjugates (Supplemental Figs. 1 and 2). Quantitative radiolabeling of the L804-IAB2MA construct in ammonium acetate buffer with ^89^Zr was achieved at ambient temperature in under 30 min at a molar activity of 9.25 MBq/nmol, which was comparable to the quantitative labeling of the DFO-IAB2MA construct under the same conditions in 2-[4-(2-hydroxyethyl)piperazin-1-yl]ethanesulfonic acid buffer (>99% determined by size-exclusion high-performance liquid chromatography). Similarly, L804-IAB2MA was radiolabeled with ^177^Lu in under 30 min at ambient temperature at a molar activity of 18.5 MBq/nmol in high radiochemical yields and purity (>99% by size-exclusion high-performance liquid chromatography). In contrast, DOTA-IAB2MA was radiolabeled with ^177^Lu for 30 min at 37°C, and only about 90% radiochemical yield could be obtained, consistent with previous reports (33). The reaction mixture was purified using Centricon (MilliporeSigma) filtration to yield ^177^Lu-DOTA-IAB2MA with purity of more than 99% and a purification yield of more than 85%. All 4 agents exhibited high stability (>95%) for 72 h at 37°C in mouse serum (Supplemental Fig. 3).

### Binding Affinity of Eu-L804-IAB2MA

When europium(III) was used as a luminescent surrogate metal ion, the binding affinity of Eu-L804-IAB2MA was measured in PSMA-positive PC3-PIP and PSMA-negative PC3-FLU prostate cancer cells by time-resolved luminescence. The Eu-L804-IAB2MA conjugate possessed high binding affinity to PSMA in PC3-PIP cells (dissociation constant, 4.6 ± 0.6 nM) (Supplemental Fig. 4). Saturation binding could not be determined for PC3-FLU cells, as total (nonspecific plus specific) and nonspecific binding were indistinguishable.

### Cell Binding and Internalization

Generally, all radiotracers were rapidly internalized after 30 min, peaking at 4 h and then plateauing at 12 h (Supplemental Fig. 5). The percentage internalization of ^89^Zr- and ^177^Lu-L804-IAB2MA was 70 ± 2.5% and 40 ± 4.1%, respectively, at 30 min and approached a maximum of 75 ± 0.9% for ^89^Zr-L804-IAB2MA and 51 ± 4.4% for ^177^Lu-L804-IAB2MA after 4 h. All radiotracers had significantly higher internalized radioactivity in PC3-PIP cells than in PC3-FLU cells (Supplemental Tables 1–4).

### Biodistribution of Radiolabeled Constructs

**FIGURE 1.**
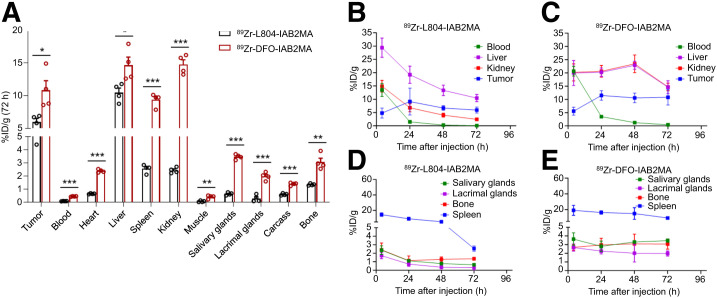
(A) Biodistribution of ^89^Zr-L804-IAB2MA and ^89^Zr-DFO-IAB2MA at 72 h after injection in PC3-PIP tumor–bearing mice. (B–E) Time–activity curves of ^89^Zr-L804-IAB2MA (B and D) and ^89^Zr-DFO-IAB2MA (C and E) in PC3-PIP tumor–bearing mice. Data are mean ± SEM (*n* = 4). *P < 0.05. **P < 0.01. ***P < 0.001.

The biodistribution of ^89^Zr-L804-IAB2MA and ^89^Zr-DFO-IAB2MA showed similar tumor localization after 4 and 24 h; however, the activity remaining in nontarget tissues was higher for ^89^Zr-DFO-IAB2MA at later time points (Fig. 1). Tumor uptake of ^89^Zr-L804-IAB2MA was 9.1 ± 2.5 %ID/g at 24 h and gradually decreased to 6.0 ± 0.6 %ID/g by 72 h after injection (Supplemental Fig. 6; Supplemental Table 5). The ^89^Zr-L804-IAB2MA agent cleared the blood after 24 h, with tumor-to-blood ratios reaching 75 ± 8.8 at 72 h (Supplemental Fig. 7). Blood clearance of ^89^Zr-L804-IAB2MA was significantly faster than that of ^89^Zr-DFO-IAB2MA (tumor-to-blood ratio, 24 ± 3.0) at 72 h after injection. At 4 h after injection, there was no significant difference in bone uptake between ^89^Zr-L804-IAB2MA (2.4 ± 0.4 %ID/g) and ^89^Zr-DFO-IAB2MA (2.7 ± 0.2 %ID/g), but significantly lower bone retention was observed for ^89^Zr-L804-IAB2MA than for ^89^Zr-DFO-IAB2MA (1.1 ± 0.27% vs 3.0 ± 0.38 %ID/g; *P* = 0.0079) at 24 h and thereafter (Supplemental Table 5). Compared with ^89^Zr-L804-IAB2MA, ^89^Zr-DFO-IAB2MA had significantly higher kidney uptake at all time points (Supplemental Fig. 6). At 72 h after injection, ^89^Zr-DFO-IAB2MA accumulation was also significantly higher in all organs examined (i.e., heart, liver, spleen, kidney, muscle, salivary glands, lacrimal glands, carcass, and bone) than that of ^89^Zr-L804-IAB2MA (Fig. 1; Supplemental Table 5), consistent with 41 ± 1.2 %ID and 69 ± 0.9 %ID (*P* < 0.0001) recovered activity, respectively, in the urine/feces (Supplemental Fig. 7).

**FIGURE 2.**
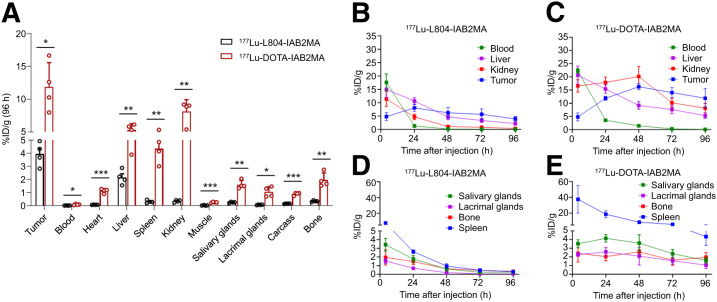
(A) Biodistribution of ^177^Lu-L804-IAB2MA and ^177^Lu-DOTA-IAB2MA at 96 h after injection in PC3-PIP tumor–bearing mice. (B–E) Time–activity curves of ^177^Lu-L804-IAB2MA (B and D) and ^177^Lu-DOTA-IAB2MA (C and E) in PC3-PIP tumor–bearing mice. Data are mean ± SEM (*n* = 4). *P < 0.05. **P < 0.01. ***P < 0.001.

In ^177^Lu biodistribution studies, significantly lower accumulation of radioactivity was observed in tumors after administration of ^177^Lu-L804-IAB2MA (8.1 ± 0.7% at 24 h and 3.9 ± 0.5 %ID/g at 96 h) than after administration of ^177^Lu-DOTA-IAB2MA (12 ± 0.4 %ID/g at 24 h, *P* = 0.0028; and 12 ± 1.9 %ID/g at 96 h, *P* = 0.0201) (Fig. 2; Supplemental Fig. 8; Supplemental Table 6). Blood clearance was significantly slower for ^177^Lu-DOTA-IAB2MA than for ^177^Lu-L804-IAB2MA (Figs. 2B and 2C; Supplemental Table 6). At 96 h after injection, ^177^Lu-DOTA-IAB2MA accumulation was significantly higher than ^177^Lu-L804-IAB2MA accumulation in tumor, blood, and all organs examined (i.e., heart, liver, spleen, kidney, muscle, salivary glands, lacrimal glands, carcass, and bone) (Fig. 2; Supplemental Fig. 9; Supplemental Table 6), consistent with 66 ± 1.1 %ID versus 92 ± 0.4 %ID (*P* < 0.0001) recovered activity, respectively, in the urine/feces (Supplemental Fig. 9). Notably, the ^177^Lu-L804-IAB2MA agent showed tumor uptake and clearance comparable to those of ^89^Zr-L804-IAB2MA (Supplemental Figs. 6 and 8). Unlabeled minibody (10 mg/kg) did not block tumor localization for any tested radiotracers in PC3-PIP tumor–bearing mice at 24 h after injection, as observed previously (21); however, significantly higher tumor uptake of all radiotracers was observed in PC3-PIP tumor–bearing mice than in PC3-FLU tumor–bearing mice (Supplemental Fig. 10).

### PET/CT and SPECT/CT Imaging

**FIGURE 3.**
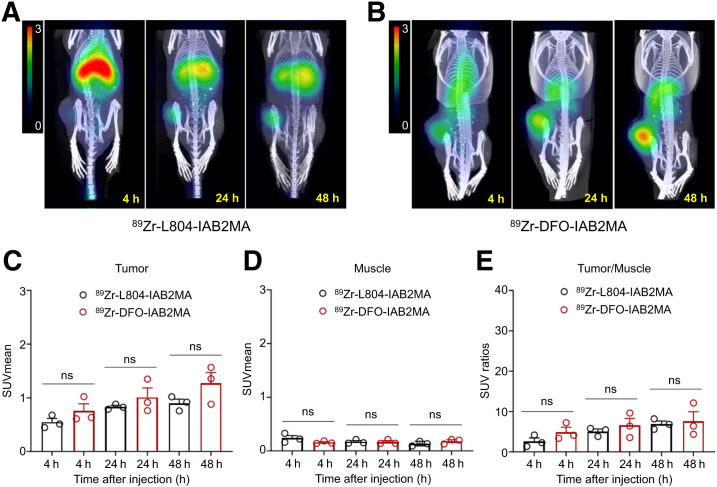
(A and B) Selected PET/CT images of ^89^Zr-L804-IAB2MA (A) and ^89^Zr-DFO-IAB2MA (B) at 4, 24, and 48 h after injection in PC3-PIP tumor–bearing mice. (C–E) Quantified SUV_mean_ for tumor (C) and muscle (D), and tumor-to-muscle SUV ratio (E). Data are mean ± SEM (*n* = 3). There were no significant differences (ns) in SUV_mean_ and SUV_ratio_ between ^89^Zr-L804-IAB2MA and ^89^Zr-DFO-IAB2MA.

**FIGURE 4.**
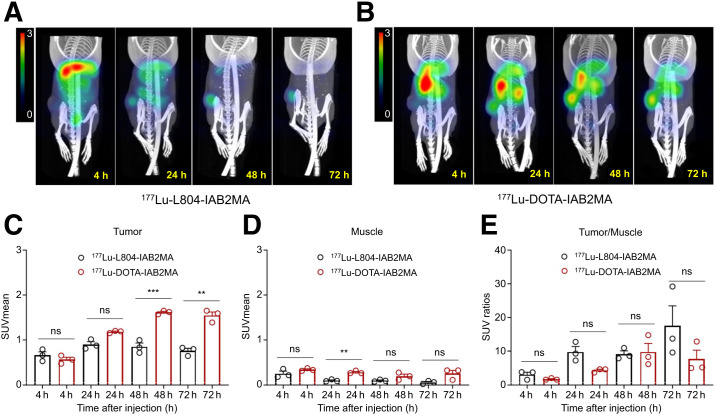
(A and B) Selected SPECT/CT images of ^177^Lu-L804-IAB2MA (A) and ^177^Lu-DOTA-IAB2MA (B) at 4, 24, 48, and 72 h after injection in PC3-PIP tumor–bearing mice. (C–E) Quantified SUV_mean_ for tumor (C) and muscle (D), and tumor-to-muscle SUV ratio (E). Data are mean ± SEM (*n* = 3). ns = not statistically significant. **P < 0.01. ***P < 0.001.

PET/CT imaging data obtained at 4, 24, and 48 h after injection (^89^Zr-L804-IAB2MA or ^89^Zr-DFO-IAB2MA) showed no significant difference in SUV_mean_ of the tumor or muscle (Fig. 3; Supplemental Table 7). SPECT/CT data at 4, 24, 48, and 72 h after injection (^177^Lu-L804-IAB2MA or ^177^Lu-DOTA-IAB2MA) showed a consistent trend toward a higher SUV for the ^177^Lu-DOTA conjugate in both tissues (Fig. 4; Supplemental Table 8). The difference in clearance rates between the 2 agents in nontarget organs in the biodistribution experiments was qualitatively corroborated by the imaging results.

### Dosimetry

Dosimetry data were computed using voxel-level–based (Imalytics) and organ-level–based (MIRDCalc) dose calculation platforms. Both platforms produced a similar pattern of absorbed doses, although MIRDCalc predicted higher absorbed doses than Imalytics. By both methods, ^177^Lu-DOTA-IAB2MA had significantly higher cumulative absorbed doses in tumors and selected organs (kidney, liver, heart, salivary glands, spleen, and muscle) than did ^177^Lu-L804-IAB2MA (Table 1; Supplemental Fig. 11). Using Imalytics, the tumor-absorbed dose from ^177^Lu-DOTA-IAB2MA (0.33 ± 0.02 Gy/MBq) was significantly higher than that from ^177^Lu-L804-IAB2MA (0.21 ± 0.02 Gy/MBq; *P* = 0.0049), and the same trend was observed with MIRDCalc (^177^Lu-DOTA-IAB2MA: 0.73 ± 0.07 Gy/MBq; ^177^Lu-L804-IAB2MA: 0.31 ± 0.03 Gy/MBq; *P* = 0.0008). The Imalytics method resulted in tumor-to-kidney ratios of 1.05 and 0.77 for ^177^Lu-L804-IAB2MA and ^177^Lu-DOTA-IAB2MA, respectively, whereas MIRDCalc gave tumor-to-kidney ratios of 1.39 and 0.78 and tumor-to-spleen ratios of 2.24 and 1.16 for ^177^Lu-L804-IAB2MA and ^177^Lu-DOTA-IAB2MA, respectively. Importantly, using MIRDCalc, we determined that the salivary dose of ^177^Lu-L804-IAB2MA was significantly lower than that of ^177^Lu-DOTA-IAB2MA (0.09 ± 0.005 vs. 0.23 ± 0.008, respectively; *P* < 0.0001), and the ratio of tumor dose to salivary gland dose was 3.6 with L804 versus 3.1 with DOTA.

**TABLE 1.** Cumulative Absorbed Doses (Gy/MBq) of ^177^Lu-L804-IAB2MA and ^177^Lu-DOTA-IAB2MA at 72 Hours After Injection and Tumor-to-Organ Ratios

Organ	^177^Lu-L804-IAB2MA	^177^Lu-DOTA-IAB2MA	*P*
Imalytics Preclinical*			
Tumor	0.21 ± 0.02	0.33 ± 0.01	0.0049
Kidney	0.20 ± 0.01	0.43 ± 0.04	0.0039
Liver	0.22 ± 0.01	0.32 ± 0.04	0.0469
Lung	0.02 ± 0.008	0.05 ± 0.02	0.4706
Muscle	0.03 ± 0.007	0.07 ± 0.005	0.0118
Tumor-to-kidney	1.05 ± 0.03	0.77 ± 0.10	0.0638
Tumor-to-liver	0.96 ± 0.07	1.05 ± 0.15	0.6471
Tumor-to-lung	13.71 ± 7.54	14.9 ± 9.24	0.9756
Tumor-to-muscle	6.74 ± 0.89	4.68 ± 0.26	0.0805
MIRDCalc^†^			
Tumor	0.31 ± 0.03	0.73 ± 0.07	0.0008
Kidney	0.22 ± 0.006	0.94 ± 0.04	<0.0001
Liver	0.51 ± 0.01	0.76 ± 0.03	0.0003
Heart	0.13 ± 0.005	0.21 ± 0.01	0.0005
Spleen	0.14 ± 0.004	0.64 ± 0.05	0.0009
Salivary glands	0.09 ± 0.005	0.23 ± 0.008	<0.0001
Tumor-to-kidney	1.39 ± 0.11	0.78 ± 0.07	0.0027
Tumor-to-liver	0.60 ± 0.06	0.96 ± 0.08	0.0130
Tumor-to-heart	2.30 ± 0.19	3.46 ± 0.26	0.0122
Tumor-to-spleen	2.24 ± 0.20	1.16 ± 0.11	0.0024
Tumor–to–salivary glands	3.64 ± 0.50	3.11 ± 0.31	0.3977

* Voxel-level–based method.

† Organ-level–based method.

Data are mean ± SEM (*n* ≥ 3).

### Efficacy of ^177^Lu-Labeled L804-IAB2MA and DOTA-IAB2MA

**FIGURE 5.**
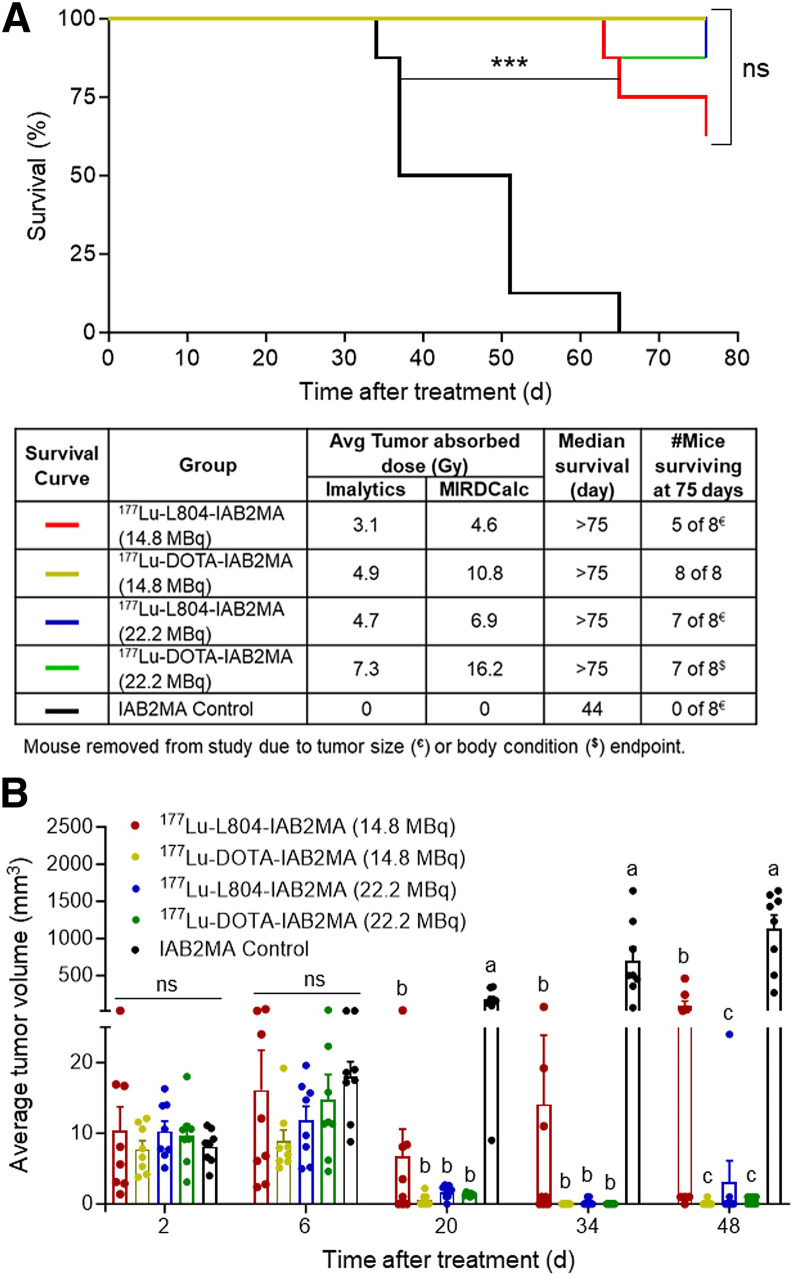
^177^Lu-L804-IAB2MA therapy in PC3-PIP tumor-bearing mice. (A) Kaplan–Meier survival curves of mice treated with ^177^Lu-L804-IAB2MA, ^177^Lu-DOTA-IAB2MA (14.8 and 22.2 MBq), or unlabeled minibody (control). (B) Average tumor volume of mice at 2, 6, 20, 34, and 48 d after treatment. Bars with different letters are significantly different (*P* < 0.05). Data are mean ± SEM.

PC3-PIP prostate cancer tumor–bearing mice treated with single doses of ^177^Lu-L804-IAB2MA (14.8 or 22.2 MBq; median survival, >75 d) exhibited significantly prolonged survival and reduced tumor volume compared with unlabeled minibody control (median survival, 44 d) (*P* < 0.0001) (Fig. 5A). No significant difference in survival was observed between groups of mice treated with ^177^Lu-L804-IAB2MA and ^177^Lu-DOTA-IAB2MA (14.8 or 22.2 MBq), although the percentage survival of the lower-dose (18.4 MBq) ^177^Lu-L804-IAB2MA group was lower than that of the DOTA agent groups (14.8 or 22.2 MBq) at the end of the study (Supplemental Table 9). Tumor volumes in mice treated with all ^177^Lu agents were significantly reduced at 20 d after treatment compared with control (*P* < 0.0001) (Fig. 5B; Supplemental Fig. 12). Tumor volumes in mice receiving ^177^Lu-L804-IAB2MA (22.2 MBq) and ^177^Lu-DOTA-IAB2MA (14.8 or 22.2 MBq) were nearly zero at 34 d after treatment. Significantly higher tumor volumes were observed for mice treated with ^177^Lu-L804-IAB2MA (14.8 MBq) than with the other ^177^Lu treatments at 48 d after treatment (*P* < 0.0001) (Fig. 5B; Supplemental Table 10). Mice receiving ^177^Lu-DOTA-IAB2MA (14.8 or 22.2 MBq) had minor tumor regrowth at 48 and 41 d after treatment, respectively (Supplemental Table 10).

**FIGURE 6.**
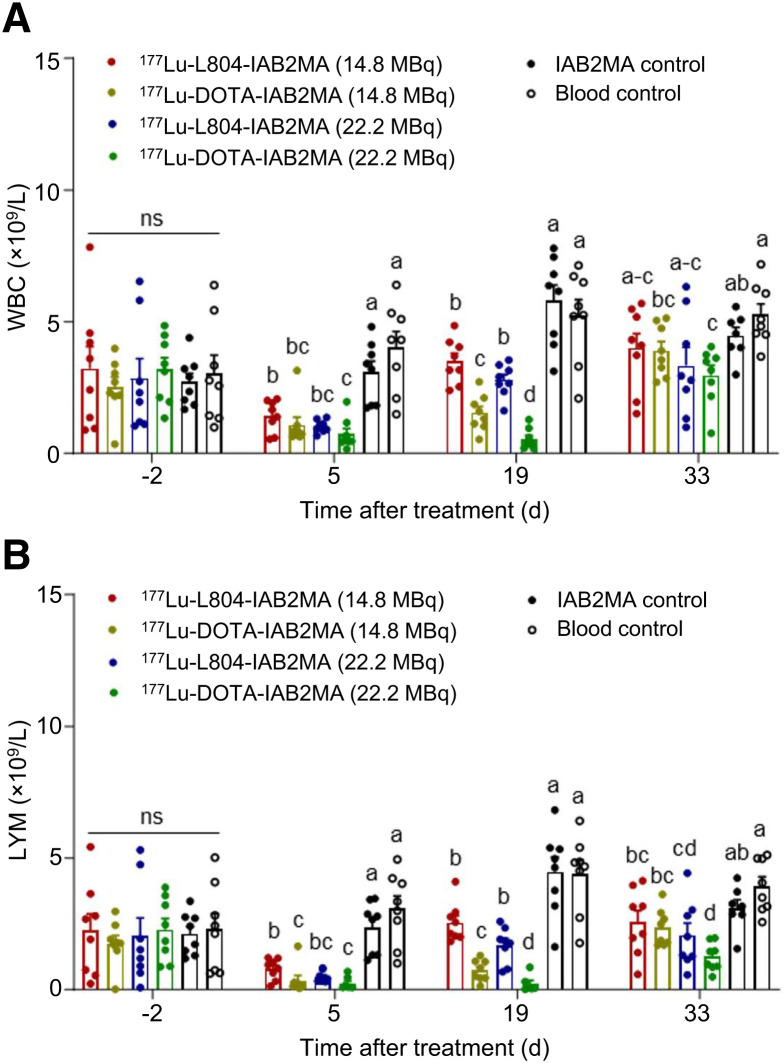
Analysis of white blood cells (WBC) (A) and lymphocytes (LYM) (B) in tail vein blood of PC3-PIP tumor–bearing mice. Bars with different letters are significantly different (*P* < 0.05). Data are mean ± SEM.

Mice treated with ^177^Lu-L804-IAB2MA exhibited faster weight recovery than those treated with ^177^Lu-DOTA-IAB2MA (Supplemental Fig. 12; Supplemental Table 11). At 2 d after treatment, mice receiving all treatments had significantly lower weight than did non–tumor-bearing mice. At 6 and 22 d after treatment, no significant difference in weight was observed between mice treated with ^177^Lu-L804-IAB2MA (14.8 or 22.2 MBq) and non–tumor-bearing mice, whereas mice receiving ^177^Lu-DOTA-IAB2MA (14.8 or 22.2 MBq) had significantly lower weight than did non–tumor-bearing mice during the study. Complete blood count analysis indicated a transient decrease in the white blood cell and lymphocyte subsets at 5 d after treatment, which recovered within 19 d for the ^177^Lu-L804-IAB2MA–treated groups and by 33 d for the ^177^Lu-DOTA-IAB2MA–treated groups (Fig. 6; Supplemental Fig. 13; Supplemental Table 12). At 19 d, the white blood cell and lymphocyte counts were significantly lower in mice receiving ^177^Lu-DOTA-IAB2MA than in mice receiving ^177^Lu-L804-IAB2MA (*P* < 0.05). One mouse treated with ^177^Lu-DOTA-IAB2MA (22.2 MBq) exhibited weight loss and very low white blood cell and lymphocyte counts and eventually died at 58 d after treatment, although there was no palpable tumor at 30 d after treatment and onward (Supplemental Fig. 14).

## DISCUSSION

Biodistribution studies comparing ^89^Zr-DFO-IAB2MA with ^89^Zr-L804-IAB2MA show statistically significant differences in tissue localization of the 2 agents at 72 h after injection (Fig. 1). A greater retention of ^89^Zr-DFO-IAB2MA in off-target tissues (e.g., spleen, kidney, heart, lacrimal/salivary glands, and bone) is consistent with reticuloendothelial system uptake and may be in part attributable to loss of ^89^Zr from DFO. Biodistribution studies comparing ^177^Lu-DOTA-IAB2MA with ^177^Lu-L804-IAB2MA also show significant differences in tissue localization at 96 h after injection (Fig. 2). Specifically, greater retention of ^177^Lu-DOTA-IAB2MA in off-target tissues (e.g., spleen, kidney, heart, lacrimal/salivary glands, and bone) is consistent with reticuloendothelial system uptake and loss of the metal ion by the DOTA chelator. The kidney uptake of ^177^Lu-DOTA-IAB2MA is similar to results reported for ^177^Lu-CHX-A″-DTPA-A11, in a study that did not pursue that agent further because of excessive kidney localization (34).

PET/SPECT and PET/CT imaging studies and dosimetry analyses generally support the conclusions of the biodistribution data (Figs. 3 and 4), particularly the faster clearance of ^177^Lu-L804-IAB2MA than of ^177^Lu-DOTA-IAB2MA. Both the biodistribution and the imaging studies indicate (at varying levels of significance) that there is greater localization of ^177^Lu-DOTA-IAB2MA and ^89^Zr-DFO-IAB2MA in PC3-PIP tumors than of the respective radiolabeled L804 conjugates. However, in both cases, the increased tumor localization is offset by the increased retention in nontarget organs, and the absorbed doses by the kidney, liver, spleen, muscle, and salivary glands are also lower for ^177^Lu-L804-IAB2MA (Table 1). At equivalent injected amounts of ^177^Lu, agents that clear more rapidly are expected to result in lower levels of tumor localization. However, after normalization for absorbed tumor dose, tumor-to-kidney and tumor-to-spleen ratios favor ^177^Lu-L804-IAB2MA.

From the absorbed doses, it is clear that these minibody agents are less dependent on renal excretion than are analogous small-molecule agents, particularly for the L804 derivative (35). Absorbed doses to the PC3-PIP tumors (0.21–0.73 Gy/MBq) were similar to those reported for ^177^Lu-PSMA-617 in C4-2 cells (0.0758 Gy/MBq) and LNCaP cells (0.594 Gy/MBq) (36,37). Estimates of kidney doses for ^177^Lu-PSMA-617 vary from 0.07 to 0.55 Gy/MBq (38), and the uncertainty derives from the fact that it is more difficult to accurately quantify critical early time points. As salivary glands are one of the dose-limiting organs for small-molecule agents (39), the avoidance of salivary gland localization by minibody-based radiotherapeutics could facilitate efficacious tumor doses at the expense of different dose-limiting organs (e.g., liver and bone marrow).

The efficacy of ^177^Lu-DOTA-IAB2MA and ^177^Lu-L804-IAB2MA after single-dose administration was compared in the PC3-PIP tumor regrowth model (Fig. 5). Although survival did not statistically differ between treatment groups, tumor growth data showed better growth control for ^177^Lu-DOTA-IAB2MA than for ^177^Lu-L804-IAB2MA, where 3 of 8 tumors regrew after treatment in the lower-dose group (14.8 MBq/mouse) and 1 tumor regrew in the higher-dose group (22.2 MBq/mouse). Tumor growth was effectively controlled in all other mice outside of the unlabeled minibody control group. When equivalent administered amounts of radioactivity were compared, the greater efficacy of ^177^Lu-DOTA-IAB2MA was consistent with the increased tumor localization from the imaging and biodistribution studies. However, there were also indications that these doses of ^177^Lu-DOTA-IAB2MA were less well tolerated, especially in the higher-dose (22.2 MBq) group, where 1 mouse of 8 failed to thrive after treatment. Complete blood count analysis showed a more pronounced depletion and slower recovery of leukocytes and lymphocytes after treatment with the DOTA agent (Fig. 6). Weight loss after treatment corroborated the hematology data, with mice recovering weight more quickly in the ^177^Lu-L804-IAB2MA groups than in the equivalent ^177^Lu-DOTA-IAB2MA treatment groups.

Taken together, we expect the maximum tolerated dose to be higher for ^177^Lu-L804-IAB2MA than for ^177^Lu-DOTA-IAB2MA, which may allow for equivalent or better tumor growth inhibition with less hematologic toxicity. If the slower clearance of ^177^Lu-DOTA-IAB2MA is due to loss of the metal ion in vivo, this effect may be more pronounced in humans because of the faster clearance generally observed in mice. Future studies would benefit from examination of further dose escalation for ^177^Lu-L804-IAB2MA and investigation in additional PSMA-positive tumor–bearing mouse models.

## CONCLUSION

The macrocyclic chelator L804 was compared with the current gold standard chelators DOTA and DFO as PSMA-directed IAB2MA minibody constructs in preclinical biodistribution, imaging, dosimetry, and efficacy studies. Although the ^89^Zr- and ^177^Lu-L804-IAB2MA conjugates showed lower tumor uptake, they generally exhibited faster nontarget organ clearance than the corresponding ^89^Zr-DFO or ^177^Lu-DOTA agents, consistent with more stable radiometal chelation. The faster clearance was also consistent with reduced salivary gland localization and reduced hematopoietic toxicity. Continued investigation of the theranostic ^89^Zr- and ^177^Lu-L804-IAB2MA agents as alternatives to current PSMA-directed radiotheranostic agents is ongoing.

## DISCLOSURE

This research was funded by the NIH Small Business Innovation Research (SBIR) Program (grant 1R43CA265652). David Tatum and Darren Magda are employed by and own stock options in Lumiphore, Inc. Fang Jia and Alessandro Mascioni are employed by ImaginAb Inc. Carolyn Anderson is on the scientific advisory board of and has funding from Lumiphore, Inc. No other potential conflict of interest relevant to this article was reported.

## Notes

KEY POINTS **QUESTION:** Is L804 a superior choice of chelator (ease of radiolabeling chemistry, biodistribution, toxicity, and efficacy) for stably complexing ^89^Zr and ^177^Lu to a PSMA-targeted IAB2MA minibody compared with the current gold standard chelators, DFO and DOTA? **PERTINENT FINDINGS:** Radiolabeling of L804-IAB2MA with ^177^Lu was achieved in more than 99% purity at room temperature in 30 min, whereas labeling of DOTA-IAB2MA required heating to 37°C, and only about 90% radiochemical yield was obtained. Although the ^89^Zr- and ^177^Lu-L804-IAB2MA conjugates showed lower tumor uptake, they exhibited faster nontarget organ clearance than did the corresponding ^89^Zr-DFO or ^177^Lu-DOTA agents, consistent with more stable radiometal chelation. This faster clearance was also consistent with reduced salivary gland localization and hematopoietic toxicity. **IMPLICATIONS FOR PATIENT CARE:** ^177^Lu-L804-IAB2MA is a promising candidate for treating PSMA-positive prostate cancer in humans, with the potential for comparable efficacy and an improved safety profile versus existing therapeutic agents.
